# Transfusion-Related Acute Lung Injury (TRALI) in Postoperative Anesthesia Care Unit (PACU) After One Unit of Platelets: A Case Report

**DOI:** 10.7759/cureus.29274

**Published:** 2022-09-17

**Authors:** Miguel E Perez-Viloria, Kalei Lopez, Fayeza Malik, Puja Yatham, Olga Lopez, Kei S Oh, Sarah Alghamdi, Guillermo Garcia

**Affiliations:** 1 Anesthesiology, Mount Sinai Medical Center, Miami, USA; 2 Anesthesiology, Florida International University, Herbert Wertheim College of Medicine, Miami, USA; 3 Medicine, Florida International University, Herbert Wertheim College of Medicine, Miami, USA; 4 Pathology, Mount Sinai Medical Center, Miami, USA

**Keywords:** postoperative complications in the pacu, platelet transfusion, anesthesia recovery period, transfusion-related acute lung injury, case reports

## Abstract

Transfusion-related acute lung injury (TRALI) following transfusion of all plasma-containing blood products is a rare but serious syndrome characterized by the acute onset of non-cardiogenic pulmonary edema with severe hypoxemia with or without symptoms of hypotension, pinkish frothy secretions, fever, and cyanosis. In this report, we present a case of a 66-year-old female with a medical history significant for hypertension, hyperlipidemia, hepatitis C, liver cirrhosis, tobacco use disorder, metastatic spindle cell carcinoma of the lung status post chemotherapy who developed TRALI after administration of one unit of platelets. Although a rare occurrence, there can be a considerable risk of TRALI following transfusion of all plasma-containing blood products and there is great importance in considering each patient’s risk factors for TRALI development prior to blood product administration.

## Introduction

Transfusion-related acute lung injury (TRALI) following transfusion of plasma-containing blood products is a rare but serious syndrome that is characterized by the acute onset of non-cardiogenic pulmonary edema with severe hypoxemia within 6 h of receiving a transfusion of any blood product [1-2]. Although resolution can occur within 48-96 h of supportive care [3]. TRALI is the leading cause of serious morbidity and mortality associated with transfused blood components reported to the United States Food and Drug Administration (FDA) [4].

General risk factors for developing TRALI vary and include a history of smoking, alcohol use disorder, liver disease, shock prior to a transfusion, systemic inflammation, and pre-existing pulmonary injury [5-7]. Transfusion-specific risk factors for TRALI involve the receipt of multiple units of blood products, and the receipt of plasma from a previously pregnant donor who has developed anti-leukocyte antibodies from alloimmunization [5, 8]. Furthermore, evidence amounts to the significant role that neutrophils play in the development of TRALI considering that patients with leukemia and lymphoma, who often present with significant neutropenia, have been reported to have a lower incidence of TRALI overall [9]. Current understanding of the triggers of TRALI suggests that the presence of cytokines and lipids in the transfused blood product activates the recipient’s pulmonary neutrophils that cause direct damage to the lungs, leading to a change in vascular permeability and the onset of pulmonary edema [10].

The three major types of TRALI that differ in their pathophysiological onset include the following: antibody-mediated, non-antibody-mediated, and possible-TRALI types [2]. Antibody-mediated TRALI is thought to develop when leukocyte-directed antibodies contained within the donor plasma of a transfused blood product bind to the cognate antigen in the transfusion-recipient, resulting in a capillary leak and subsequent lung injury [11]. Despite this conceptualization of injury, antibodies are not identified in the transfused product in nearly 20% of TRALI cases regardless of the use of sensitive assays. Non-antibody mediated TRALI is postulated to be due to exposure to some other type of biological reactive molecules contained within the plasma of the transfused blood product which ultimately serves as the inciting factor leading to lung injury [12]. The third major type is possible-TRALI which includes the same criteria as TRALI in addition to the risk factors for ARDS such as sepsis and pneumonia [13]. Interestingly, a prominent pretransfusion elevation in inflammatory cytokines such as IL-8 is expected whereas the presence of anti-leukocyte antibodies is rare thus contributing to the unique pathophysiology behind possible-TRALI [14].

The expected clinical presentation of TRALI involves rapid onset of dyspnea, tachypnea, fever, pink-colored secretions with a frothy consistency, hypotension or hypertension, cyanosis, and transient leukopenia [4, 15]. A diagnosis of TRALI relies on the exclusion of other causes of acute pulmonary edema following transfusion which includes but is not limited to sepsis from pneumonia, anaphylaxis, cardiogenic pulmonary edema, and transfusion-associated circulatory overload (TACO) [16]. In particular, distinguishing between TRALI and TACO often can be quite difficult given their overlapping symptomatology. Whereas TACO typically presents as pulmonary cardiogenic edema, TRALI can be expected to manifest as non-cardiogenic pulmonary edema and identified using the key diagnostic features found in the table further described in the discussion [17]. In general, diagnostic criteria for TRALI includes (1) acute onset hypoxemia within 6 h of transfusion; (2) a PAO2/FiO2 ratio of less than 300 mmHg or oxygen saturation of less than 90% on room air; (3) bilateral pulmonary infiltrates on chest radiograph; and (4) pulmonary artery wedge pressure < 18 mmHg with no evidence of left atrial hypertension [18].

This case report serves to highlight the caution that must be taken with the perioperative administration of a single unit of platelets due to the rare risk of transfusion-related injury manifesting as TRALI. After receiving only one unit of platelets, while undergoing a major surgical procedure, the 66-year-old patient succumbed to the complications related to TRALI. Currently, there are no other reports in the literature of TRALI development after the administration of only one unit of platelets. Overall, despite TRALI development being rare after sole administration of an all-plasma blood product, the significant morbidity and mortality associated with TRALI make consideration of predisposing factors of transfusion-related injury essential in the management of patients receiving such products in the perioperative setting.

## Case presentation

A 66-year-old female with a medical history significant for hypertension, hyperlipidemia, hepatitis C, liver cirrhosis, tobacco use disorder, metastatic spindle cell carcinoma of the lung status post carboplatin/pemetrexed/pembrolizumab chemotherapy, on pemetrexed/pembrolizumab for maintenance, recurrent pleural effusion, and recent positive positron emission tomography (PET) scan for enlarged mediastinal lymph nodes suspicious for malignancy. The patient presented to the operating room for fiberoptic bronchoscopy with ultrasound biopsy, right video-assisted thoracoscopy, robotic-assisted drainage of right pleural effusion, right lower lobe wedge resection, and talc pleurodesis. Pre-operative work-up was unremarkable except for a low platelet count of 55,000 (N: 150-450 10^3/uL) and a chest X-ray (CXR) indicative of trace small bilateral pleural effusions with associated bibasilar atelectasis. In the holding area, two large bore IVs and a radial arterial line were placed. Induction and intubation were uneventful while total IV anesthesia (propofol and remifentanil) was used for maintenance of anesthesia. Endobronchial ultrasound was performed to identify the enlarged right hilar mediastinal lymph nodes. Transbronchial needle biopsy of these enlarged mediastinal lymph nodes and additional lymph nodes at this level were performed using a 19 Vizi-Shot needle (Olympus, PA, USA). A pathological review of these samples was deemed benign.

**Figure 1:**
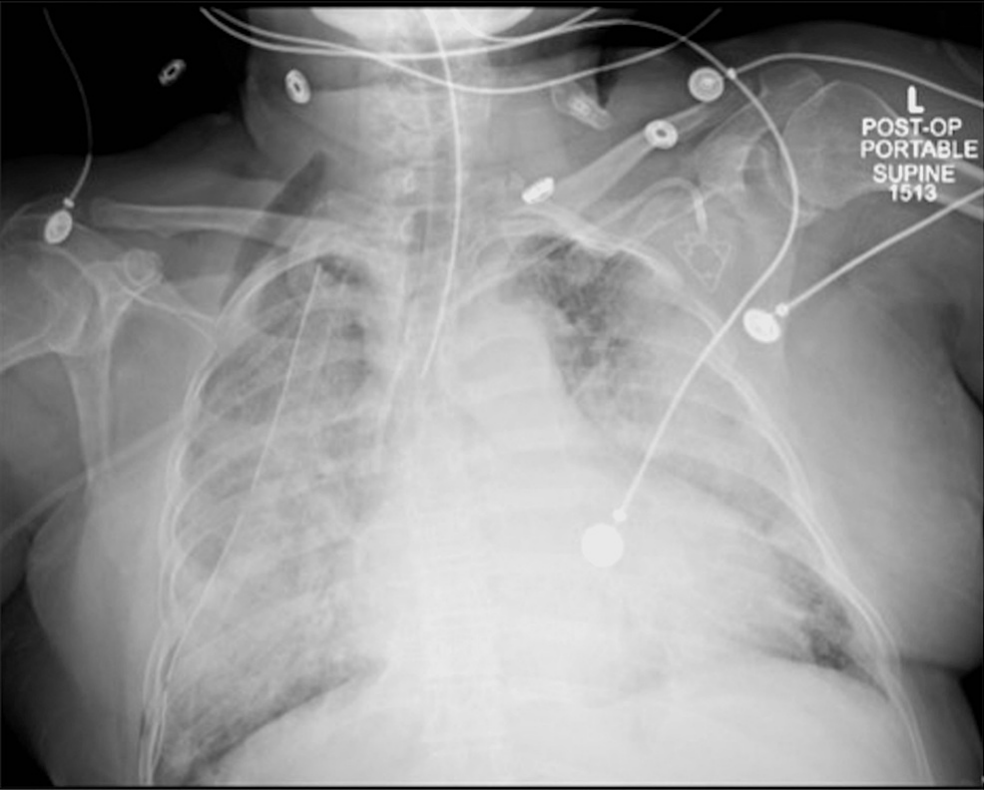
Chest X-ray displaying diffuse bilateral interstitial and airspace opacities suggestive of pulmonary edema.

**Figure 2:**
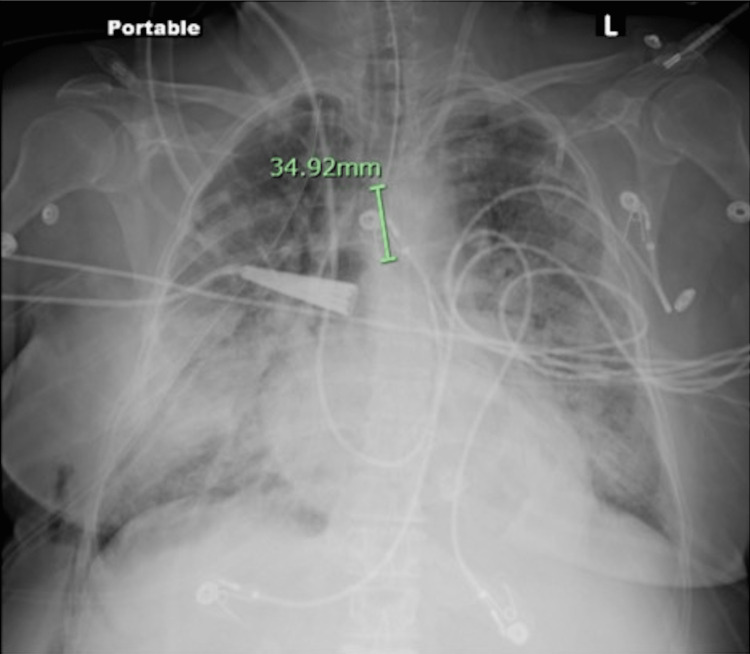
Chest X-ray displaying worsening infiltrates bilaterally likely due to worsening pulmonary edema.

The decision was made to proceed with a right robot-assisted thoracoscopy instead of thoracentesis due to the large amounts of blood visualized during biopsy and the low preoperative platelet count. The ninth intercostal space at the right posterior axillary line was entered, and a moderate-sized effusion of serous fluid was visualized with the presence of fibrin in the pleural cavity causing loculations. This fluid was suctioned, and the remaining two robotic ports were placed at the nipple line and mid-axillary line of the right seventh intercostal space. At the level of the right lower lobe, near the inferior pulmonary ligament, the lung presented a multilobular appearance with induration that would be expected to be found with a lung nodule. An Endo-GIA 60 purple load (Medtronic, NH, USA) was placed, samples were taken for pathological review, and talc pleurodesis was performed. Intraoperatively, the surgeon observed significant microvascular bleeding. In response to the surgeon’s observation and preoperative thrombocytopenia, a bag of platelets was transfused. In addition to the platelets, the patient received 700 mL of IV crystalloids throughout the case. At the conclusion of the surgery, neuromuscular blockade was reversed and the patient was found to be hemodynamically stable, achieving adequate tidal volumes, and following commands appropriately. The patient was extubated and was able to maintain an unobstructed upper airway without assistance. Within a few minutes, the patient became increasingly tachypneic with a respiratory rate of 35 breaths per minute and showed a rapid progression to hypoxia with a SaO2 of 87%, requiring immediate reintubation while still in the operating room. Auscultation revealed rales in all lung fields. A CXR demonstrated interval development of diffuse, bilateral interstitial infiltrates suggestive of pulmonary edema (Figure 1). The patient was transferred to the postoperative anesthesia care unit (PACU) intubated on mechanical ventilation. A workup was done including labs (Table 1). Swan-Ganz catheter (Edwards Lifesciences, CA, USA) placement showed a pulmonary artery pressure of 26/15 mmHg with a central venous pressure of 8 mmHg. A repeat CXR showed worsening infiltrates bilaterally likely due to worsening pulmonary edema (Figure 2). A transthoracic echocardiogram revealed moderately impaired left ventricular (LV) systolic function with a left ventricular ejection fraction (LVEF) of 40%. The right ventricular size and systolic function were normal. Troponin levels were within normal limits. Given these findings, the patient’s acute decompensation was determined to be consistent with TRALI. The patient was then transferred to the surgical intensive care unit (SICU) where she continued to deteriorate, eventually progressing to multiorgan failure with expiration on postoperative day six. Further testing by the blood center failed to identify anti-human leukocyte antigen (HLA) Class I & II, and anti-human neutrophil antigen (HNA) antibodies in the subsequent sample from the implicated donor. While this result rules out the immunogenic cause of TRALI, one may still consider a probable TRALI of non-immunogenic etiology. The case report presented is in accordance with Case Report Guidelines (CARE) (see the figure in the Appendix).

**Table 1:** Laboratory values in the PACU. PACU,  post anesthesia care unit; WBC, white blood count; CO2, carbon dioxide; uL, microliter; mmol, milimole per liter; mg, miligram; L,  liter; dL, deciliter

Variable	Result	Reference values
WBC	19.2	4.8-10.8 10^3/uL
Platelets	132,000	150-450 10^3/uL
CO2	19.0	21.0-32.0 mmol/L
Chloride	110	98-107 mmol/L
Creatinine	1.21	0.55-1.02 mg/dL
Calcium	7.6	8.5-10.1 mg/dL

## Discussion

The TRALI was first described in 1983 by Popovsky et al. when following five patients who developed signs of fever, acute hypoxemia, hypotension, respiratory distress, and non-cardiogenic pulmonary edema within four hours of blood product transfusion [19]. TRALI currently is the leading cause of transfusion-related mortality and warrants careful approach and frequent monitoring during blood transfusions, especially in those who are more vulnerable for acquiring TRALI [4]. Some of the risk factors for developing TRALI include patients with malignancies, shock, positive intravascular fluid balance, systemic inflammation, high peak airway pressure during mechanical ventilation, low interleukin-10 levels, and those undergoing liver and cardiovascular surgery [20]. It is estimated that nearly one in every 5000 blood transfusions is complicated by the development of TRALI [2].

Understanding of the pathophysiology behind TRALI remains incomplete, though it is contemporarily described as a process requiring two distinct events in succession. First, the transfusion recipient would have a certain baseline amount of inflammation present which sensitizes resident neutrophils in the lungs [21]. The presence of inflammation serves to increase the overall risk of transfusion-related injury followed by the second event which is the activation of neutrophils by antibodies, lipids, extracellular vesicles, or signaling molecules present in the transfused product [21]. This neutrophilic activation leads to even greater levels of localized inflammation, further damaging lung tissue and pulmonary blood vessel linings which ultimately leads to non-cardiogenic pulmonary edema and respiratory distress [21]. This is why every case of TRALI should be reported to the blood bank so that donors who might potentially cause the same reaction, in future recipients, are identified [22].

Transfusion-associated circulatory overload is the second leading cause of transfusion-related mortality and can easily be confused with TRALI given their similar course of symptomatology following transfusion [23]. Whereas both TACO and TRALI feature acute respiratory distress secondary to pulmonary edema, the absence of signs of circulatory overload distinguishes TRALI from the other [24]. In this case, the patient’s symptoms were consistent with the expected presentation of TRALI given their normal central venous pressure, normal pulmonary artery wedge pressure, absence of S3 on auscultation, near normal LV function, and no signs of peripheral edema on physical exam (Table 2).

**Table 2:** Diagnostic characteristics of TRALI. TRALI, transfusion related acute lung injury; SPO2, oxygen saturation; PaO2, arterial partial pressure of oxygen; FiO2, fraction of inspired oxygen; pg, picogram; mL, milliliter; BNP, B-type natriuretic peptide

TRALI characteristics [17]	
Diagnostic feature	Quantitative or qualitative measure
Acute presentation of respiratory distress	Onset within 6 h of blood transfusion
Hypoxemia	SPO2 <90% or PaO2/FiO2 < 300 mmHg on room air
Pulmonary edema	Chest X-ray displaying bilateral infiltrates
Risk factors	Sepsis, pneumonia, acute pancreatitis, aspiration, multiple trauma
Increased end-diastolic volumes in critically ill patients	BNP >250 pg/mL or pre-/posttransfusion BNP ratio >1.5

The differential diagnosis for patients presenting with TRALI-like symptoms should include other complications that originate from transfusion of blood products. Allergic and anaphylactic transfusion reactions also present with respiratory distress and hypoxia due to bronchospasm and laryngeal edema following transfusion of blood products [15-16]. The absence of wheezing, urticarial rash, and hypotension help rule out allergy and anaphylaxis as possible diagnoses [15-16]. Negative pressure pulmonary edema (NPPE) is an uncommon complication of anesthesia usually resulting from laryngospasm after extubation (approximately 0.1%). The most common risk factors include young age, male sex, and head or neck surgery. NPPE is an example of a noncardiogenic pulmonary edema that could resemble TRALI. The mechanism of NPPE is similar to hydrostatic pulmonary edema as observed in patients suffering from congestive heart failure or volume overload states [25-28]. The absence of upper airway obstruction rules out this etiology.

**Figure 3:**
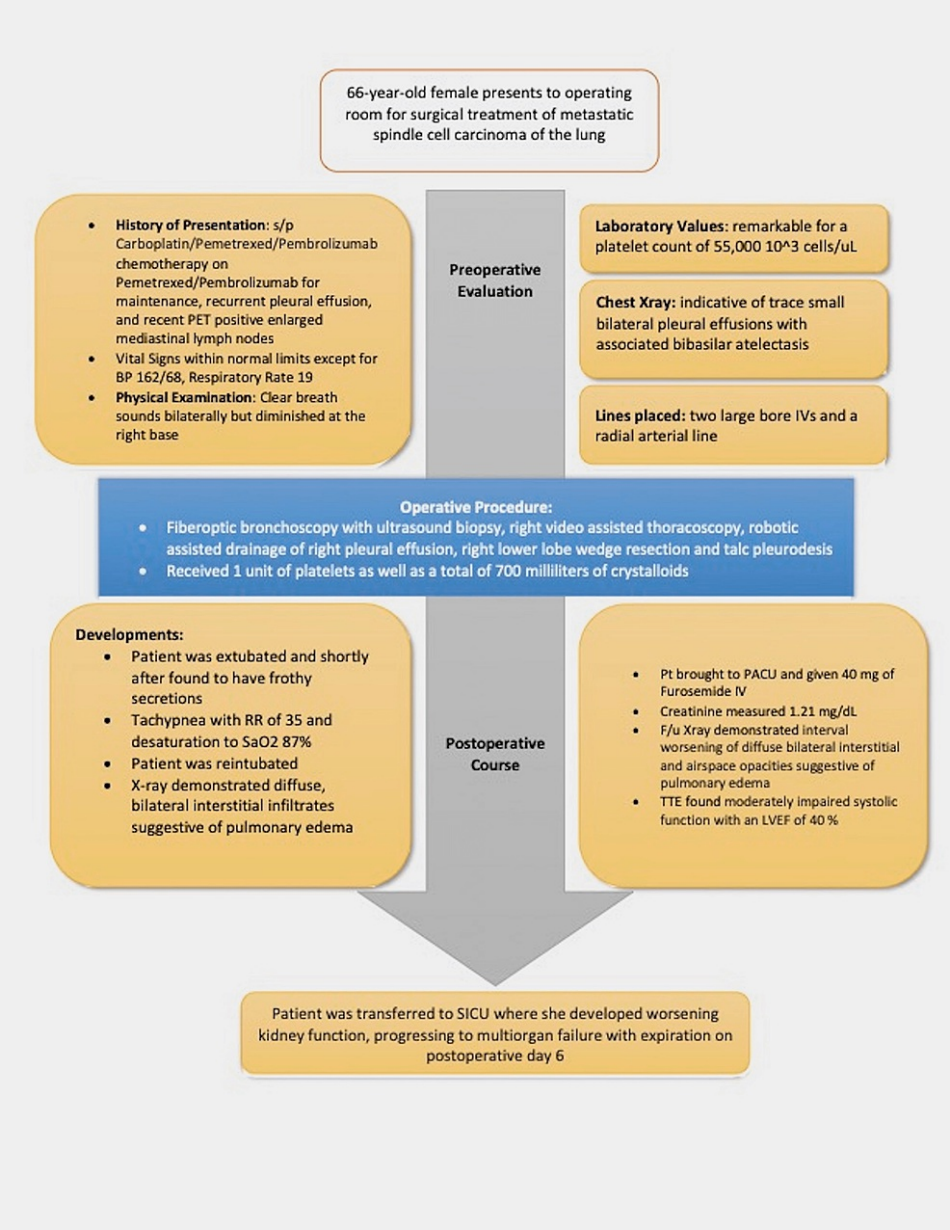
Timeline highlighting the case events. Image credits: Kalei Lopez

This case report highlights the importance of identifying predisposing risk factors prior to the utilization of blood products. This patient’s risk factors included malignancy, liver disease, history of smoking, lung disease, and alcohol use disorder which predisposed her to developing TRALI. In this patient, TRALI manifested with dyspnea, tachypnea, and desaturation. TACO was ruled out by transthoracic echocardiography and Swan-Ganz monitoring showing no signs of fluid overload. Despite administering furosemide, the symptoms of the patient did not improve which is consistent with the usual course of TRALI in that nearly 70% of TRALI patients face worsening pulmonary symptoms, requiring them to be placed on mechanical ventilation. Further, the mechanism behind the hemodynamic instability in this patient is still not clear. The normal pulmonary artery pressure of 25/16 mmHg and central venous pressure (CVP) argue against fluid overload and myocardial ischemia (Figure 3).

## Conclusions

Anesthesiologists are important in the recognition and initial treatment of TRALI, given their association with another contributor to TRALI development. Although TRALI has traditionally been understood to be a two-hit process, there is a possible third contributor to the process as well. Like the two-hit model, patient factors serve as the first hit which predisposes them to TRALI when receiving the transfusion which acts as the second hit. Additionally, the third hit is surgery, which is known to increase inflammatory markers, thereby increasing the chances of perioperative TRALI. Given their role in supervising many transfusions, anesthesiologists should screen for high-risk patients preoperatively. Overall, the best way to reduce TRALI cases is to minimize the number of unnecessary blood transfusions administered to patients. Considering that TRALI does not have a specific treatment option, it is essential that emphasis be placed on prevention. This case is unique in that administration of only one unit of platelets led to the development of TRALI. In conclusion, this case report highlights the importance of assessing the risk of TRALI when a blood transfusion is contemplated during surgery.

## Figures and Tables

**Figure 4:**
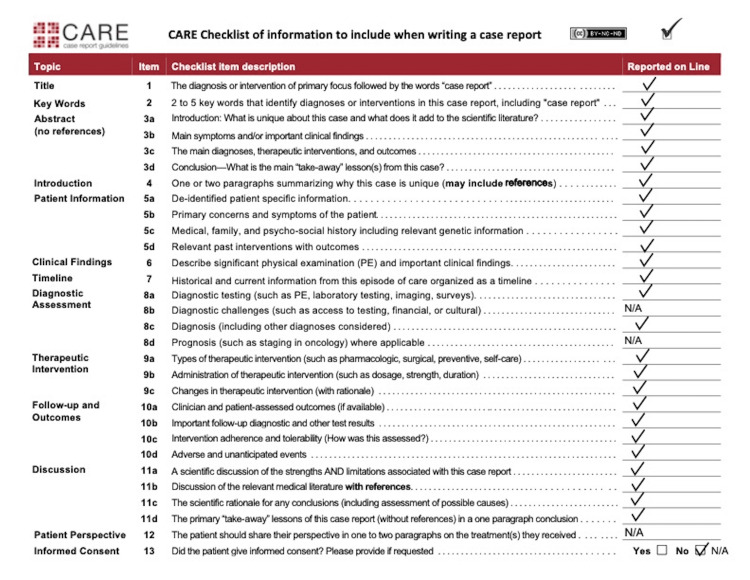
CARE checklist. The case report presented is in accordance with Case Report Guidelines (CARE).
